# Successful Delayed Repair of a Penile Fracture due to COVID‐19 Infection

**DOI:** 10.1155/criu/9751872

**Published:** 2026-06-09

**Authors:** Darren William Rodgers, Keith Pace

**Affiliations:** ^1^ Urology Unit, Mater Dei Hospital, Msida, Malta, materdei.com.br

## Abstract

**Background:**

Penile fracture is a rare condition characterised by disruption of the tunica albuginea typically caused by blunt trauma to a tumescent penis. It is a urological emergency that requires prompt diagnosis with a view to urgent treatment. The current global consensus on management of penile fractures is urgent surgical exploration and repair of the tunical defect. This is recommended with a view to minimise the risk of long‐term functional outcomes, mostly relating to sexual dysfunction. However, the lack of high quality data on ‘immediate’ versus ‘delayed’ surgical repair groups in terms of long‐term outcomes means that the exact timing of surgical repair is still debatable.

**Case Presentation:**

A 50‐year‐old healthy sexually active male presented after a snapping sound during sexual intercourse 90 minutes before, followed by detumescence and penile pain. A tunical breach in the left proximal penile shaft was diagnosed clinically and confirmed on emergency ultrasound. The patient tested positive for COVID‐19 and, given the perioperative risk of morbidity and mortality, it was decided to delay surgical repair. 10 days after the injury, following a negative COVID‐19 test, he successfully underwent circumferential degloving, circumcision and closure of the tunical defect. Upon review at 4 and 20 months post‐op, there was no scarring or penile curvature, and he was having normal painless erections and penetrative sexual intercourse.

**Conclusions:**

This is a case of early presentation of penile fracture in a healthy sexually active male, where surgical repair was delayed by 10 days because of COVID‐19 infection, with positive short and medium term outcomes. It reinforces the idea that penile fracture patients who present late should still be offered surgery and that delaying surgery to optimise a patient′s condition may be an acceptable approach. However, more research is needed into timing of surgical repair and its effect on long‐term functional outcomes.

## 1. Introduction

Penile fracture is a rare urological emergency with potentially significant biopsychosocial sequelae. It occurs when there is traumatic disruption to the tunica albuginea, which surrounds the corpora cavernosa, typically caused by blunt trauma to a tumescent penis. The commonest cause is sexual intercourse but it may also be caused by forceful masturbation, non‐sexual physical trauma and cultural practices such as ‘taqaandan’, which involve manually bending the distal erect penis to achieve quicker detumescence [1].

The classic presentation is an audible ‘pop’ at the time of injury followed by rapid detumescence and penile pain, swelling and bruising. Penile deformity may also be evident and the presence of gross haematuria could be suggestive of a concomitant urethral injury. Penile fractures are usually diagnosed only with a history and physical examination, however imaging can be used in unclear cases. Ultrasound, MRI and cavernosography may all be used to identify tunical defects radiologically, which can also be useful for surgical planning. If urethral injury is suspected, retrograde urethrogram and flexible cystoscopy are useful diagnostic tools [2].

Upon diagnosis, prompt surgical exploration, haematoma evacuation and tunical repair is the most widely recommended treatment option. A subcoronal circumferential degloving approach is commonly used. However, a longitudinal incision over the defect is also widely performed, with no direct comparison of long‐term outcomes between the two techniques ever studied [3].

Regarding management of penile fractures, a historical shift has occurred from conservative to surgical management, with the latter being immediate and not delayed, in an attempt to reduce the risk of long‐term complications mostly relating to sexual dysfunction. We present a case of penile fracture in a 50‐year‐old healthy sexually active male where surgical repair was delayed by 10 days because the patient was infected with COVID‐19, with successful postoperative outcomes.

## 2. Case Presentation

A 50‐year‐old healthy male presented to the emergency department in April 2022 following an incident during sexual intercourse with his female partner approximately 90 minutes before. He reported a sudden snapping sound followed by detumescence, penile pain and swelling. He was able to pass urine freely and denied gross haematuria.

On examination, he was noted to have gross penile swelling, left‐sided abnormal angulation at the proximal third of the penile shaft and bluish discolouration mostly on the left side. On palpation, a tunical breach was suspected in the left proximal third of the penile shaft corresponding to the bruising pattern. The patient was haemodynamically stable and afebrile. An emergency ultrasound was performed which confirmed a breach of the tunica albuginea in the proximal third of the left side of the penis, around 1 cm in size, with an associated haematoma.

As per local policy at the time, the patient underwent a rapid antigen COVID‐19 test, which resulted positive. In view of this result, a benefit‐risk assessment was performed by two senior urology consultants together with the on‐call anaesthesiologist and a shared decision with the patient was reached to delay surgery. The patient was discharged home where he self‐isolated and was covered with a course of oral co‐amoxiclav.

Following a negative COVID‐19 test, he underwent surgical repair 10 days after sustaining his penile fracture. On preoperative examination, the swelling and haematoma of the penile shaft had significantly decreased, and the defect of the tunica albuginea was more easily palpable. Circumferential degloving, circumcision, evacuation of corporeal haematoma and closure of the tunical defect were performed under general anaesthesia. The surgery was technically favourable with minimal inflammatory changes and a clearly identifiable tunical defect. The tunica and corpora cavernosa were healthy, with no infective changes or necrosis observed. No perioperative complications were noted, he was kept overnight for observation and discharged home the following day.

The patient was followed up 4 days post‐op for a wound review where no issues were encountered. Subsequently, he was assessed at 4 and 20 months after his surgery. His surgical wound had healed up nicely without scarring or penile curvature. He was having normal erections and penetrative sexual intercourse without difficulty or pain. His postoperative International Index of Erectile Function (IIEF‐5) score was 22/25, considered as no erectile dysfunction, with individual item scores of 3, 5, 5, 5 and 4 for Questions 1–5, respectively. Even though an IIEF‐5 was not calculated pre‐op, the patient did not subjectively report any changes in erectile function preinjury and postinjury. There was also no evidence of any psychosocial complications.

## 3. Discussion

The global consensus on management of penile fractures has shifted towards a surgical approach. Upon clinical suspicion or diagnosis of a penile fracture, closure of the tunica albuginea in a timely manner is currently the most widely recommended option. The American Urological Association advises ‘prompt’ surgical exploration and repair performed ‘at the time of presentation’ [4]. The British Association of Urological Surgeons specifies a time frame of 24 hours within which surgical repair of a penile fracture is recommended [5]. In the presence of associated urethral injury, however, surgical repair should be performed ‘as soon as possible’. The European Association of Urology similarly recommends ‘immediate’ repair, referencing Kominsky et al. [3], a systematic review which concluded that surgical repair should be performed in the first 24 hours [2].

Surgical as opposed to conservative management has clearly been proven to reduce the risk of long‐term complications, including erectile dysfunction, penile curvature, development of nodules/plaques and painful erections. However, the issues around the timing of the surgical repair are more debatable. The lack of high‐powered research on immediate versus delayed repair as well as the heterogeneity in the ‘delayed repair’ definition means there is currently no clear consensus on timing.

A meta‐analysis on management of penile fractures by Amer et al. looked into different complications in the immediate versus delayed surgical repair as a subgroup analysis [1]. On pooled analysis, development of penile curvature was significantly higher in the delayed group (*p* = 0.0004, *I*2 = 46*%*) and so were overall complications, unspecified (*p* < 0.001, *I*2 = 7*%*). However, crucially, erectile dysfunction rates were not significantly different between the two groups (*p* = 0.59, *I*2 = 0*%*). The authors, however, did not define ‘immediate’ and ‘delayed’, and time between injury and repair was not specifically accounted for.

Similarly, Hardesty et al. looked into penile fracture management strategies with a focus on patients with delayed presentation (> 24 hours following injury) [6]. The systematic review identified 20 studies that met eligibility criteria and in the 7 studies which directly compared outcomes in early versus late repair, 4 studies showed worse outcomes in the delayed group (including erectile dysfunction, penile curvature and painful intercourse). The other 3 studies showed no significant difference in terms of outcomes between the two groups. However, similar to Amer et al. 2016, a major limitation cited by the authors was the lack of uniformity in the definitions of early and late repair between the different studies. Heterogeneity was also encountered when assessing most of the functional outcomes, especially penile curvature, which was generally measured through highly subjective means. Additionally, in the 4 studies which had worse outcomes in the delayed group, follow up was relatively short (between 3 and 12 months) whereas in the 3 studies that showed no significant difference, follow‐up was between 6 and 28.8 months, possibly making the latter results more reliable. Another systematic review by Wong et al. included 12 studies, again taking the delayed surgical repair to be more than 24 hours from time of injury [7]. Similar to Amer et al. 2016, penile curvature favoured immediate repair whereas erectile dysfunction and tunical scar formation did not.

In light of this and the above studies, the difference in functional outcomes in the post‐24 hour ‘delayed repair’ population in comparison to the ‘immediate repair’ group is not very conclusive, especially when it comes to erectile dysfunction. Therefore, this reinforces the idea that patients who present late should still be offered surgical repair as the likelihood of long‐term positive functional outcomes is still favourable.

Our case describes successful delayed repair 10 days after onset of symptoms. Generally, there is very little data on delayed repair of penile fractures beyond 7 days post‐injury. Naraynsingh et al. reported three cases where repair was intentionally performed after 12, 10 and 7 days, at which point in all cases, penile swelling had resolved allowing better visualisation of the fracture site [8]. Direct repair was chosen in favour of degloving, with all three patients having ‘normal painless erections’ after 18, 14 and 8 months respectively. A small observational study by Nasser and Mostafa assessed 24 patients presenting after 24 hours of injury who underwent 7–12 days of conservative treatment followed by surgical repair under local anaesthesia [9]. Sexual activity was regained normally after 4–6 weeks in all cases.

Just like in our case, in these studies patients were allowed a delay in their surgical repair to optimise their condition and given the positive results, this strategy may be an acceptable approach to management. In our case, the justification for delayed surgery related to the increased perioperative risks of COVID‐19 in a period of relative uncertainty. It was noted that surgery under general anaesthesia in a COVID‐19 positive patient, even though asymptomatic, carried a significant risk of peri‐operative morbidity and mortality. These risks include postoperative pneumonia, respiratory failure and thrombotic complications [10, 11]. Following the benefit‐risk assessment, it was determined that the risk of morbidity and mortality associated with surgery under general anaesthesia outweighed the risk of sexual dysfunction associated with delaying surgical repair of the penile fracture. This was thoroughly discussed with the patient who also agreed with delaying surgery.

In terms of timing of delayed repairs, a difference in clinically significant outcomes has not yet been clearly demonstrated. What is definitely clear is that more high‐powered research is required in order to optimise management of penile fractures, especially those who present late. More studies need to look into timing of surgical repair relative to onset of injury, with the ‘post‐24 hour group’ divided into different time frames allowing for direct comparison in terms of long‐term outcomes. In this way, outcomes of Day 2 post‐injury patients, who may have gross penile swelling complicating surgery, can be compared with those of patients 10 days post‐injury, who may be in a more optimal condition to undergo surgery.

## 4. Conclusion

A 50‐year‐old healthy sexually active male with an early presentation of penile fracture had surgical repair delayed by 10 days because of COVID‐19 infection, with successful short and medium term outcomes. Prompt surgical repair is recommended by urological scientific societies globally. However, this case reinforces the idea that patients with a late presentation of penile fracture should still be offered surgical repair and that delaying surgery by a few days in order to optimise a patient′s condition may be an acceptable approach. A decision to delay surgery should always be made in liaison with the anaesthesiology team based on a careful benefit‐risk assessment, and shared decision‐making with the patient is also key. More high‐powered research is needed into the specific timing of surgical repair and its effect on long‐term outcomes.

## Funding

No funding was received for this manuscript.

## Disclosure

All authors have read and approved the final version of the manuscript. Mr. Darren William Rodgers and Mr. Keith Pace had full access to all of the data in this study and take complete responsibility for the integrity of the data and the accuracy of the data analysis.

## Consent

Written informed consent was given by the patient.

## Conflicts of Interest

The authors declare no conflicts of interest.

## Data Availability

The clinical data used to support the findings of this case report are included within the article.
